# A Few Points in the Diagnosis of Ulcers—Running Sores

**Published:** 1901-09

**Authors:** Thomas H. Manley

**Affiliations:** Professor of Surgery in the New York School of Clinical-Medicine, Visiting Surgeon to Harlem Hospital


### A FEW POINTS IN THE DIAGNOSIS OF ULCERS—RUNNING SORES.

By THOMAS H. MANLEY, Ph.D., M.D.,

Professor of Surgery in the New York School of Clinical-Medicine, Visiting Surgeon to Harlem Hospital.

The science and the art of diagnosis have made great strides during the past twenty years. Pathology and diagnosis are indessolubly bound together; the former implies a knowledge of disordered physiological phenomena, with the structural or organic changes resulting, an exact appreciation and interpretation of these changes constitutes *diagnosis*.

*The recognition of the various types of ulcers* and their causation, is by no means a simple matter.

Mistakes and blunders in this direction are responsible for large numbers of surgical mutilations, the needless torture and sacrifice of organs, and, alas! it may be said, of even life itself.

Ulcers depending on multitudinous causes, must necessarily present a great diversity of characters. In their gross anatomical elements and their clinical phenomena, they are all essentially alike, *i. e.*, they involve a solution of continuity in the soft or hard parts, they rapidly spread and as rapidly shrink, they burn, bleed and are painful.

Space here will not permit of anything like a full amplification of the subject and hence but a few brief notes will be submitted on the more common varieties.

ULCERS DEPENDANT ON SCROFULA OR TEBUCLE, OR EPITHELIAL-HYPERPLASIA CANSER ON GUMMA-SYPHILIS.

Except varicose ulcer of the leg, the scourge of the poor, the most common type we see, in civil life, is dependent on scrofula in the young and terboculosis in the adult.

Cancerous ulcers is seldom or never seen until adult years and after middle life, and these are special areas which it is especially prone to seize on.

The character of the pain and the peculiar fetor which its discharges emit are its most distinguishing clinical features.

Luetic ulcer on the mucous surfaces, or that consecutive to gummy growths, is not so commonly seen. With these venereal lesions may be added the chancrous ulcers, or the chancroid.

These, when extra genital are rare, and derive their greatest interest from the possibility of confounding them with those of a more innocent origin.

PERIPHERAL AND INTERNAL OR ORGANIC ULCER.

Surface ulceration of the cutaneous, is the most common and simple of diagnosis. The most serious difficulties are met with when the deep structures or the outlets of the body are involved, as:

1. The lips, mouth or pharynx.
2. The anus or rectum.

3. The labia or the vagina—in the female.

In this class we derive the largest share of information from:

1. The history of the case, the patient's age, ancestry, occupation and habits.

2. From the patient's general condition and the clinical phenomena.

3. A digital and occular examination.

4. From a micro-histological examination of the morbid tissues.

5. From a microscopical examination of the discharges, cultures or inocculations.

6. Systematic medication over an ample period to determine the "therapeutic test."

Hasty, unskillful or inadequate diagnosis here may be most disastrous in its consequences.

The practitioner should always approach these cases fortified by a broad knowledge, and if not well seasoned in practice, should seek the aid of those of larger surgical experience.

He should never accept as final the opinion of any colleague in the matter of diagnosis, because all are fallible and prone to commit oversights, or mistakes, with this phase of medical science.

The above is but a "summary" of a large topic.

